# Effect of miRNA-200b on the proliferation and apoptosis of cervical cancer cells by targeting RhoA

**DOI:** 10.1515/med-2020-0147

**Published:** 2020-10-08

**Authors:** Lijie He, Jing Wang, Dandan Chang, Dandan Lv, Haina Li, Heqiang Feng

**Affiliations:** Departments of T Lymphocyte Subpopulation, Tianjin Fifth Central Hospital, Tianjin, 300450, People’s Republic of China; Departments of Immunologic Fuction, Tianjin Fifth Central Hospital, Tianjin, 300450, People’s Republic of China; Department of Biochemistry, Tianjin Fifth Central Hospital, 41 Zhejiang Road, Tianjin, 300450, People’s Republic of China

**Keywords:** miRNA-200b, RhoA, cervical cancer, proliferation, apoptosis

## Abstract

**Objective:**

This article aims to investigate the effect of miRNA-200b on the proliferation and apoptosis of cervical cancer cells by targeting RhoA.

**Methods:**

HeLa cells of cervical cancer were divided into five groups: blank control group, negative control group (miRNA-200b mimic NC), miRNA-200b mimic group, RhoA-negative control group, and RhoA overexpression group. Cells were collected 48 h after transfection. The expression levels of miRNA-200b were detected by RT-PCR. Target relationship between miRNA-200b and RhoA was verified by the dual-luciferase reporter assay. RhoA mRNA and protein expression were detected by western blot and RT-PCR methods. Flow cytometry was used to detect the apoptosis of cells in each group, and the CCK8 method was used to detect the proliferation of cells in each group. The mRNA and protein expression of Bax and cyclin D1 were detected by RT-PCR and western blot.

**Results:**

The results of the dual luciferase reporter assay showed that RhoA was the target gene of microRNA 200b. Compared with the blank control group and the miRNA-200b mimic-NC group, the proportion of apoptotic cells increased significantly in the miRNA-200b mimic group, and the proliferation of cells was inhibited (*P* < 0.05). After overexpression of RhoA, the percentage of apoptotic cells decreased and the ability of cell proliferation increased significantly (*P* < 0.05).

**Conclusion:**

miRNA-200b can inhibit the proliferation and promote the apoptosis of cervical cancer cells by targeting the RhoA gene.

## 1 Introduction

Cervical cancer has become the second most prevalent malignancy among young and middle-aged women in China, accounting for more than 28% of cases worldwide. In recent years, the incidence and mortality of cervical cancer have been increasing significantly in China [1]. MicroRNAs (miRNAs) are endogenous single-stranded small RNAs that can play an important role in the development and progression of human tumors through posttranslational modifications [2]. A previous study has reported that miRNAs are closely related to the proliferation, apoptosis, and invasion of cervical cancer cells and are considered as new targets for the diagnosis, treatment, and prognostic evaluation of cervical cancer [3]. MiRNA-200 is a family of miRNAs that inhibit tumors, including miRNA-200a, miRNA-200b, and miRNA-200c, and is involved in tumor development and progression [4]. A previous study has found that miRNA-200b was abnormally expressed in epithelial ovarian cancer, and plasma miRNA-200b could be used as a molecular marker for early diagnosis of epithelial ovarian cancer [5]. RhoA is a small G protein with GTPase activity that regulates the biological function of cells. Studies have found that RhoA is overexpressed in a variety of cancer tissues and cancer cells and is closely related to tumor invasion and metastasis [6,7]. Bax is a member of the Bcl-2 family and plays a role in promoting apoptosis. Overexpression of Bax can not only cause spontaneous apoptosis of many cells but also increase apoptosis caused by many factors [8]. Cyclin D1 is abnormally overexpressed in a variety of malignant tumors, which can promote the proliferation of tumor cells [9]. Currently, there are few reports about the effect of mirna-200b and RhoA protein on the proliferation and apoptosis of cervical cancer cells. However, there have been few reports on the effects of miRNA-200b and RhoA proteins on the proliferation and apoptosis of cervical cancer cells. Therefore, in the present study, the targeting relationship between miRNA-200b and RhoA protein was verified, and its role and possible mechanism of action in cervical cancer cell proliferation and apoptosis were investigated, providing a new target for the treatment of clinical cervical cancer.

## 2 Materials and methods

### 2.1 Reagents and main instruments

#### 2.1.1 Test reagents

HeLa cells of human cervical cancer were purchased from the cell bank of Shanghai Institute of Biochemistry and Cell Biology, Chinese Academy of Sciences; fetal calf serum was purchased from Gibco, USA; Lipofectamine2000 was purchased from Invitrogen of the United States; RIPA lysate, BCA protein quantitation kits, ECL chemiluminescent solution, and CCK8 kits were purchased from Beyotime Biotechnology Co., Ltd, China; rabbit anti-human RhoA monoclonal antibody (No. EPR18134), rabbit anti-human Cyclin D1 monoclonal antibody (No. EPR2241), rabbit anti-human Bax monoclonal antibody (No. EPR18283), rabbit anti-human VEGF monoclonal antibody (No. Y103), and mouse anti-human TGF-β1 monoclonal antibody (No. Y103) TB21 were purchased from Abcam, USA; horseradish peroxidase-labeled goat anti-rabbit IgG and goat anti-mouse IgG (secondary antibodies) were purchased from ZSBIO, China.

#### 2.1.2 Main instruments

CO_2_ incubator was purchased from SHELDON, USA; BD FACS Calibur flow cytometry was purchased from BD, USA; real-time fluorescent quantitative PCR instrument was purchased from Eppendorf, Germany; hypothermic high-speed centrifuge was purchased from Heraeus, Germany; automatic enzyme-labeling instrument, electrophoresis apparatus, and transfer box were purchased from Bio-Rad, USA; shaker (TS-8) was purchased from Shanghai Precision Instrument Manufacturing Co., Ltd, China; chemiluminescence imaging system was purchased from GE, USA.

## 3 Methods

### 3.1 Cell culture and transfection

HeLa cells of human cervical cancer were inoculated in RPMI-1640 culture medium (including 10% fetal calf serum) and cultured in 5% CO_2_ incubator at 37°C. Cells in the logarithmic phase were collected for the experiment. HeLa cells were divided into five groups according to different treatment methods, namely, blank control group, miRNA-200b mimic-NC group, miRNA-200b mimic group, RhoA NC group, and RhoA overexpression group. HeLa cells of human cervical cancer were inoculated on a six-well plate at a concentration of 1 × 106 cells/mL with overnight incubation. Two tubes were taken. 45 µL DMEM was added to one tube and mixed with 5 µL miRNA-200b mimics, overexpression NC group, RhoA blank control plasmid, blank control plasmid, and RhoA overexpression plasmid, respectively; 45 µL DMEM was added to another tube and mixed with 5 µL Lipofectamine2000. The two tubes were mixed and placed for 5 min and then inoculated on a six-well plate after fusion for 20 min to establish miRNA-200b mimic group, miRNA-200b mimic-NC group, RhoA NC group, and RhoA overexpression group, and the untransfected cells were considered as the blank control group. The cells of each group were collected after transfection and used for follow-up experiment.

### 3.2 Detection of miRNA-200b expression of cells in each group by RT-PCR

Total RNA of HeLa cells in each group was extracted by the Trizol method, and cDNA was obtained after reverse transcription. miRNA-200b took U6 as an internal parameter and adopted SYBR Green I real-time fluorescent quantitative PCR technique to detect the miRNA-200b expression level of cells in each group. The primers were designed and synthesized by Sangon Biotech (Shanghai) Co., Ltd, which were designed as follows: miRNA-200b upstream 5′-GGGGTAATACTGCCTGGT-3′, downstream 5′-TGCGTGTCGTGGCGTC-3′; U6 upstream 5′-GCTTCGGCAGCACATATACTAAAAT-3′, downstream 5′-CGCTTCACGAATTTGCGTGTCAT-3′. The reaction conditions are pre-denaturation at 95°C for 3 min, denaturation at 95°C for 10 s, and annealing at 60°C for 30 s, which were repeated for 40 times. Each sample was tested for three times. The relative expression of miRNA-200b of each sample in each group was calculated according to the formula (2^−ΔΔCt^ method).

### 3.3 Dual luciferase reporter assay

Databases such as bioinformatics software TargetScan were adopted to analyze the target gene of miRNA-200b, and the results showed that there are binding sites of miRNA-200b at the 3′ end of RhoA gene, suggesting that RhoA is the direct target gene of miRNA-200b. This result was verified by the dual luciferase reporter assay, and RhoA 3′UTR containing the binding sites of miRNA-200b and RhoA 3’UTR series containing the binding site mutants of miRNA-200b were amplified and inserted into the luciferase reporter gene vector to construct wild-type RhoA-Wt and mutant RhoA-Mut recombinant plasmids. HeLa cells were inoculated on a 96-well plate and cultured for 24 h at 37°C. Then, RhoA-Wt recombinant plasmids, RhoA-Mut plasmids, and miRNA-200b mimic plasmids (sequence: 5′-UAAUACUGCCUGGUAAUGAUGA-3) or mimic-N (sequence: 5′-UUCUCCGAACGUGUCACGUTT) were co-transfected into the cells, respectively, which were placed in a 37°C incubator and cultured for 48 h. The cells were collected and lysed and analyzed using dual luciferase reporter genes.

### 3.4 Detection of RhoA mRNA and protein expression of cells in each group by western blot and RT-PCR

The cells in each group that were transfected for 48 h were collected, and RIPA cell lysate was added to extract the total protein in the cells on ice. Tthen, BCA protein assay kits were adopted to quantify the protein. The protein sample was mixed with the loading buffer, which was heated in a boiling water bath for denaturation. The same amount of the denatured protein sample was added to the loading well, and SDS-PAGE gel electrophoresis was performed. After the protein was separated, it was transferred to the PVDF membrane and sealed in 5% skim milk with the mass concentration of 5% for 1 h. After the membrane was washed with TBST, RhoA primary antibody (diluted 1:1,500) was added and hybridized overnight at 4°C. After the membrane was washed with TBST, horseradish peroxidase-labeled secondary antibodies (diluted at 1:3,000) were added and hybridized at room temperature for 1 h. After the membrane was washed with TBST, proteins were detected by chemiluminescence (ECL) and imaged and photographed in a dark room with β-actin as the internal standard protein. Image J analysis software was adopted to calculate the relative expression level of RhoA protein of cells in each group. The RhoA mRNA detection method refers to the steps in Section 1.2.2. The primers were designed as follows: RhoA upstream 5′-TGATTGTTGGTGATGGAGCCT-3′, downstream 5′-ACTCTACCTGCTTTCCATCCAC-3′; β-actin upstream 5′-GATTGGAATCTGGCTACT-3′, downstream 5′-TAGGGCTGAAGCACAGGG-3′.

### 3.5 Detection of apoptosis in each group by flow cytometry

After transfection, the cells of each group were digested with 0.25% trypsin without EDTA to prepare a single-cell suspension, which was centrifuged and washed with PBS for three times. The cells were resuspended in pre-chilled 1× binding buffer to make the density approximately 1 × 10^6^ cells/mL. 1.25 µL Annexin V-FITC was added and mixed gently, which reacted for 15 min at room temperature in the dark. The cells were resuspended in 0.5 mL of pre-chilled 1× binding buffer. 10 µL propidium iodide was added, and the rate of apoptosis was detected by flow cytometry. The experiment was repeated for three times, and the average was taken.

### 3.6 Detection of cell proliferation in each group by CCK8 method

The cells of each group were collected and inoculated on a 96-well plate at a concentration of 2 × 10^3^ cells/well, which was placed in a 37°C incubator for routine culture. After 48 h of transfection, 10% culture medium of CCK8 detection reagent was added to each well and was cultured in a conventional incubator for 2 h. The optical density (OD) value of cells in each well at 450 nm wavelength was measured using the enzyme-labeling instrument. The experiment was repeated for three times.

### 3.7 Detection of protein and mRNA expression

Detection of protein and mRNA expression of Bax, Cyclin D1, VEGF, and TGF-β1 was done by Western blot and RT-PCR with specific detection methods referring to Sections 1.2.2 and 1.2.4. The primers of Bax, Cyclin D1, VEGF, and TGF-β1 were designed as follows: Bax upstream 5′-GTGGAGGAGCTCTTCAGGGA-3′, downstream 5′-AGGCACCCAGGGTGATGCAA-3′; Cyclin D1 upstream 5′-GCCGAATTCATGGAACACCAGCT-3′, downstream 5′-TGCACCTGTAGACTGAG-CTCGC-3′; VEGF upstream 5′-ATGAACTTTCTGCTGTCTTGG-3′; downstream 5′-TCACCGCCTCGGC-TTGTCACA-3′; TGF-β1 upstream 5′-CTACTACGCCAAAGAAGTCACC-3′, downstream 5′-GAAATCGG-CCCTGTACCGTCTCT-3′.

### 3.8 Statistical methods

SPSS22.0 statistical software was adopted for data analysis. All experimental data showed a normal distribution, and the results were expressed by mean ± standard deviation (\overline{x}\pm s). One-factor analysis of variance was used for the statistical analysis of multi-sample experimental results. There was a significant difference (*P* < 0.05).

## 4 Results

### 4.1 Comparison of miRNA-200b expression level in HeLa cell after the transfection of miRNA-200b

**Figure 1:**
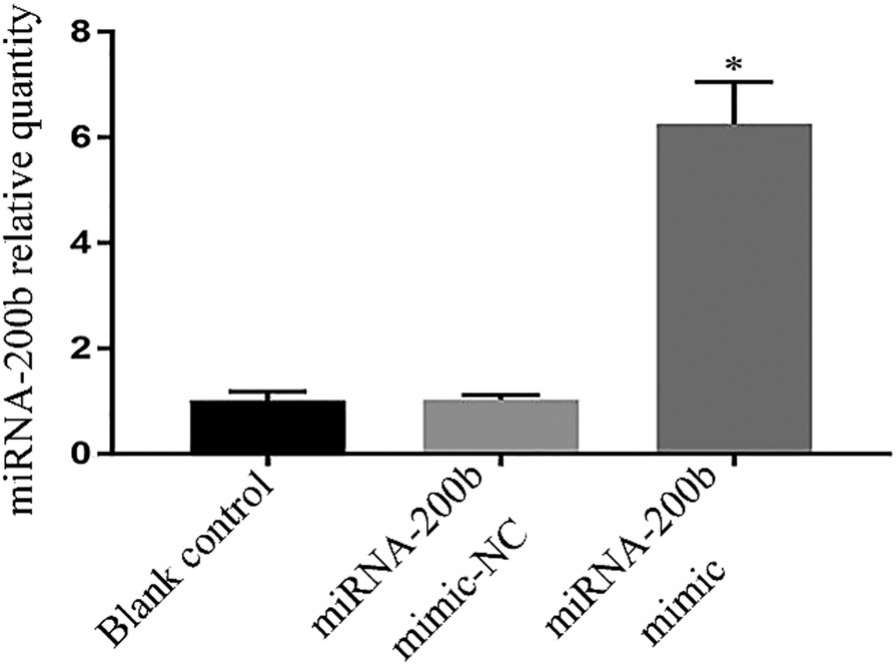
Comparison of miRNA-200b expression levels in each group. Note: compared with blank control group and mimic-NC group, ^*^ *P* < 0.05.

Compared with the blank control group and the mimic-NC group, the miRNA-200b mimic group showed a significant increase in the miRNA-200b expression level, with *P* values of 0.000 and 0.000, respectively, and there was a significant difference (*P* < 0.05). Compared with the miRNA-200b expression level in the mimic-NC group, the *P* value in the blank control group was 0.961, and there was no significant difference, as shown in Figure 1.

### 4.2 Verification of miRNA-200b’s targeted regulation to RhoA

**Figure 2:**
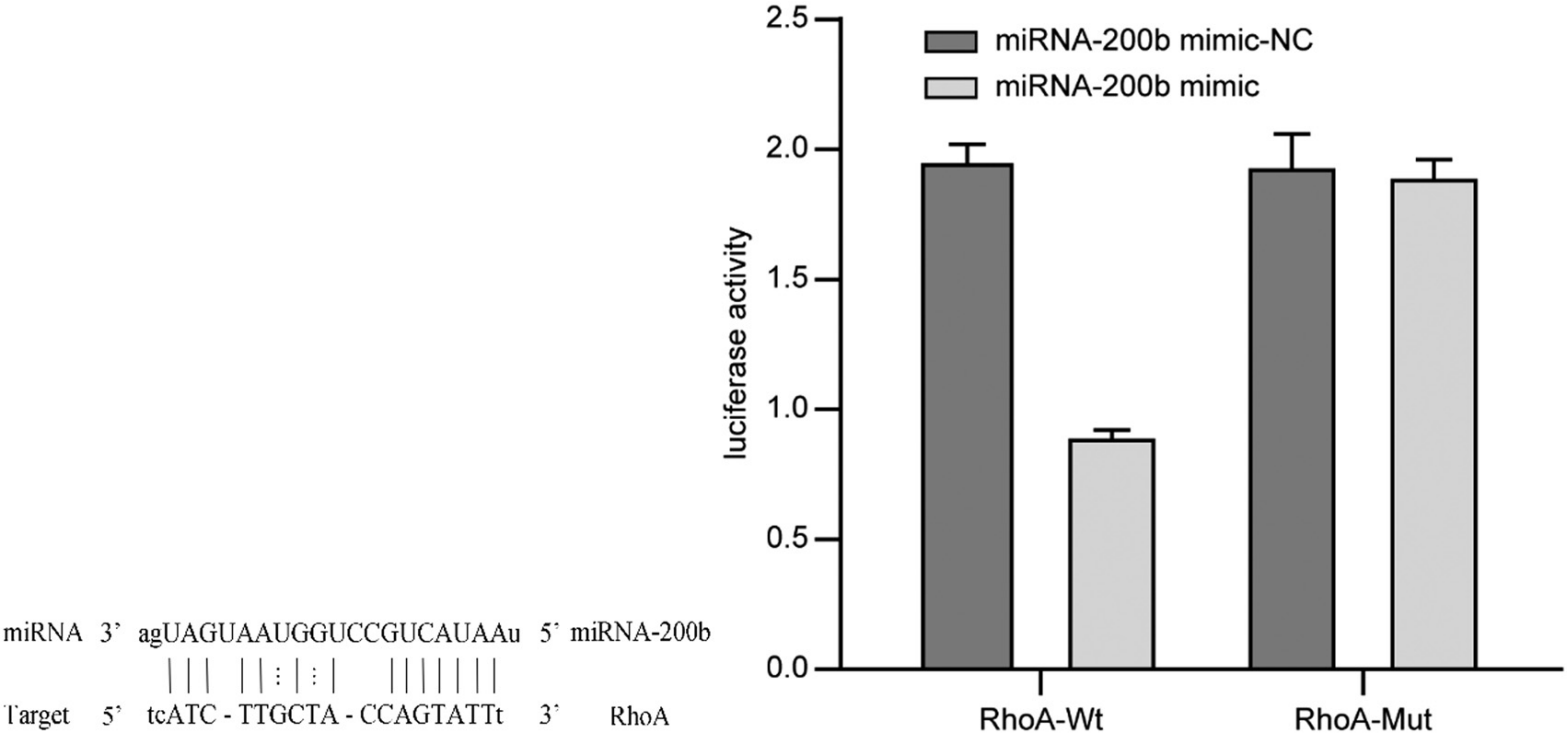
Conservative analysis of miRNA-200b binding sites and activity analysis of luciferase. Note: compared with mimic-NC group, ^*^ *P* < 0.05.

The results of the dual luciferase reporter assay showed that miRNA-200b and RhoA gene 3′UTR have complementary binding sites, and miRNA-200b and RhoA can be combined in a targeted way. The luciferase activity test results showed that the luciferase activity of miRNA-200b mimic cells transfected with the wild-type RhoA gene expression vector WT-3′UTR and then transfected with mimic-NC were significantly reduced; in RhoA-Mut, there is no obvious inhibitory effect on the luciferase activity of HeLa cell that RhoA gene expression vector mutant-3′UTR was transfected, and then, miRNA-200b mimic cells were transfected, as shown in Figure 2.

### 4.3 Comparison of RhoA protein and mRNA expression levels of cells in each group

**Figure 3:**
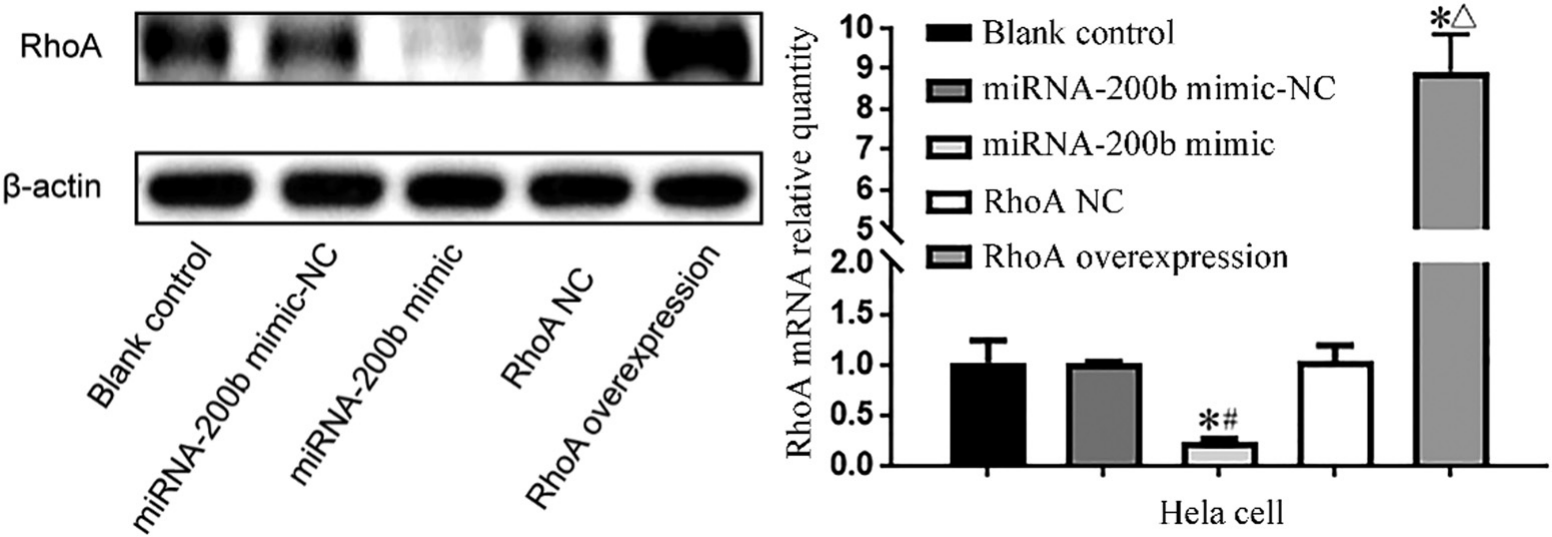
RhoA protein and mRNA expression levels of Hela cells in each group. Note: compared with blank control group,^*^ *P* < 0.05; compared with miRNA-200b mimic-NC group, ^#^ *P* < 0.05; and compared with RhoA NC group, ^Δ^ *P* < 0.05.

Compared with the blank control group and the mimic-NC group, the RhoA gene expression level in the miRNA-200b mimic group decreased significantly, with the *P* values of 0.048 and 0.048, respectively. Compared with the RhoA NC group, the miRNA-200b mimic group showed a significant increase in RhoA expression after overexpression of RhoA, with a *P* value of 0.044, and there was a significant difference (*P* < 0.05). Compared with the RhoA expression in the mimic-NC group and RhoA NC group, the *P* value in the blank control group was 1.000 and 0.948, respectively, and there was no significant difference (*P* > 0.05), as shown in Figure 3.

### 4.4 Cell proliferation and apoptosis levels in each group

**Figure 4:**
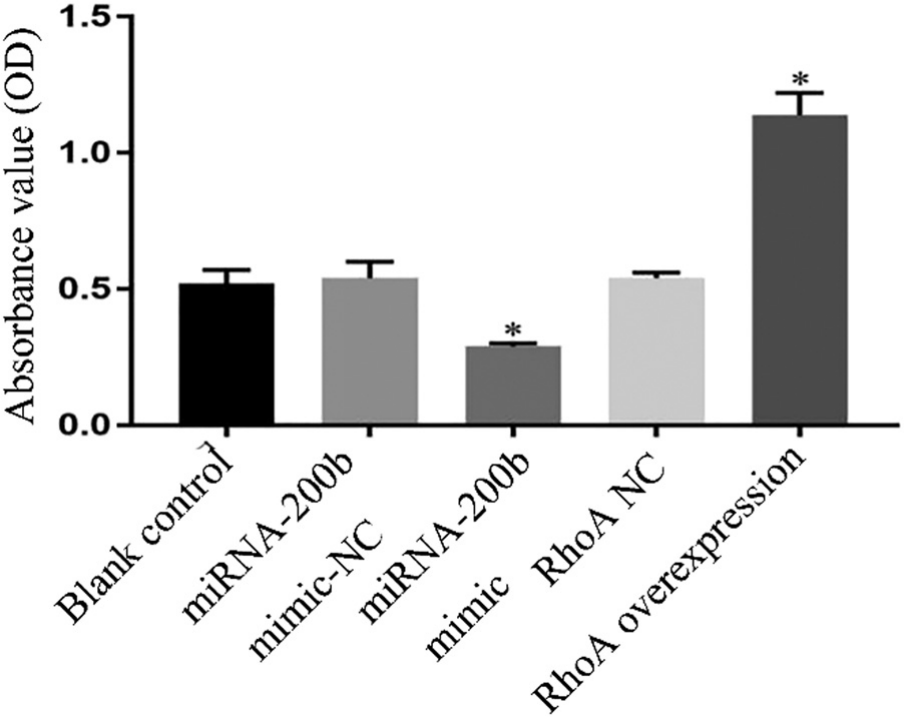
Comparison of proliferation ability after transfection in each group. Note: compared with blank control group and mimic-NC group, ^*^ *P* < 0.05.

**Figure 5:**
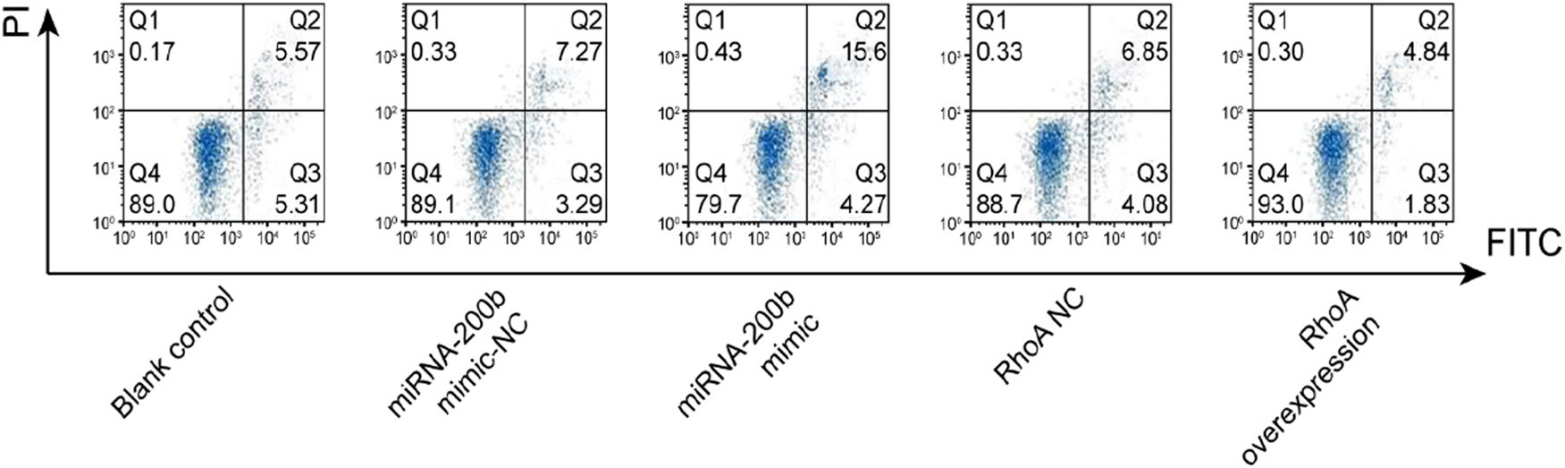
Apoptosis ability of Hela cells after transfection in each group.

Compared with the blank control group and mimic-NC group, the miRNA-200b mimic group showed a significant increase in the proportion of cell apoptosis and inhibited cell proliferation, and the *P* values were 0.001 and 0.001, respectively; in the RhoA overexpression group, the proportion of cell apoptosis decreased and the cell proliferation ability was significantly enhanced and the *P* values were 0.001 and 0.001, respectively, and there was a significant difference (*P* < 0.05); the apoptosis and the proliferation ability in the blank control group were compared with those in the mimic-NC group and the RhoA NC group, and the results showed that the *P* values were 0.987 and 0.965, respectively, and there was no significant difference (*P* > 0.05). These were shown in Figures 4 and 5.

### 4.5 Comparison of expression levels of cell proliferation-related genes and apoptosis-related genes in each group

**Figure 6:**
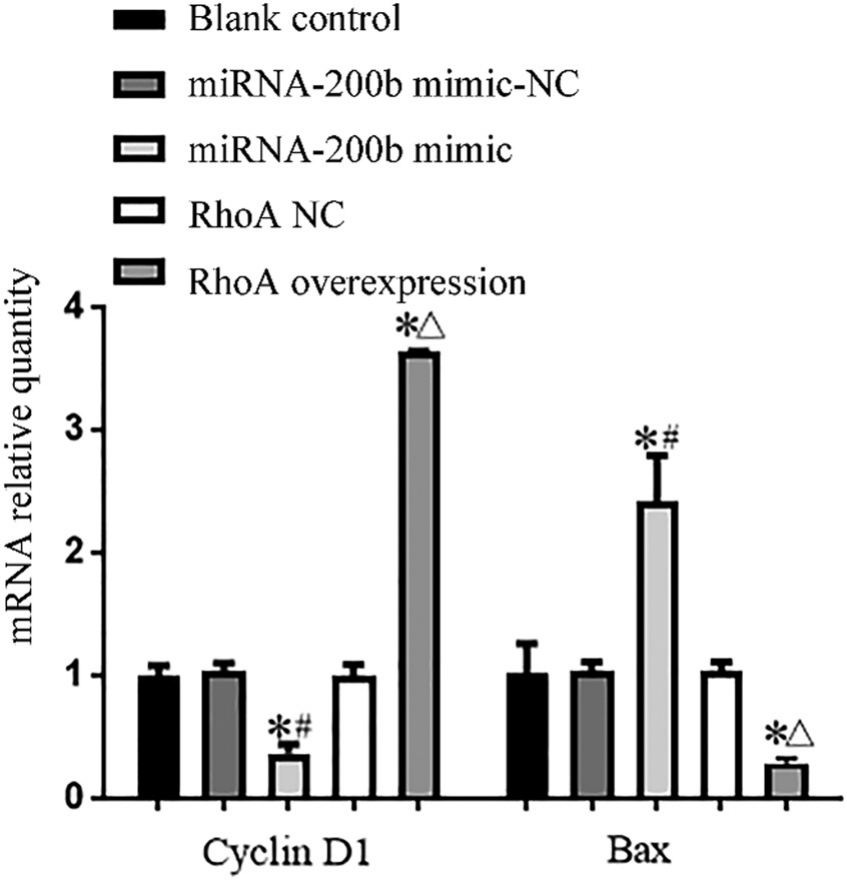
Comparison of cyclin D1 and Bax gene mRNA expression levels in each group. Note: compared with blank control group, ^*^ *P* < 0.05; compared with mimic-NC group, ^#^ *P* < 0.05; and compared with RhoA NC group, ^Δ^ *P* < 0.05.

**Figure 7:**
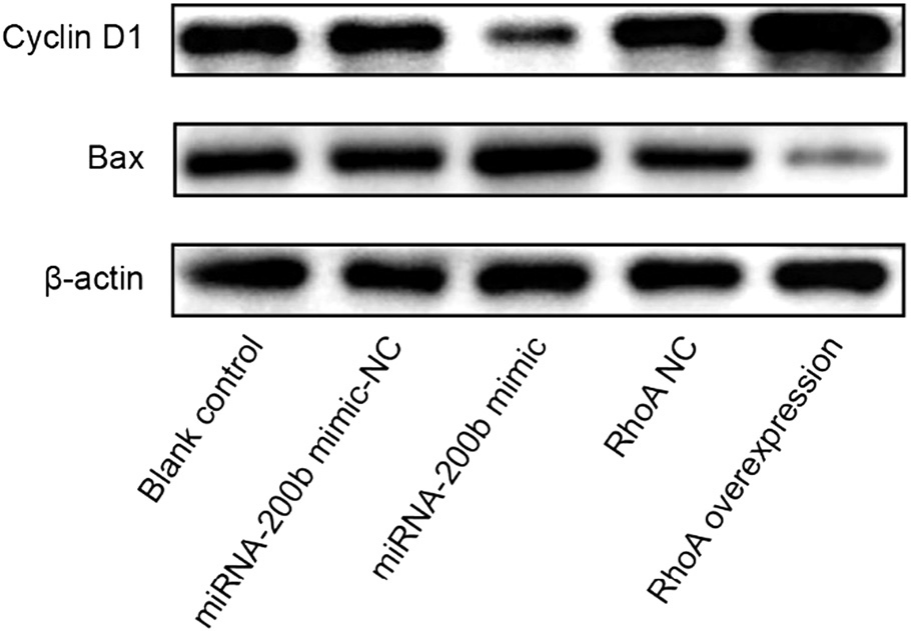
Protein expression levels of cell Cyclin D1 and Bax genes in each group.

Compared with the blank control group and the mimic-NC group, the miRNA-200b mimic group had significantly increased Bax gene mRNA and protein expression levels, with the *P* values of 0.000 and 0.000, respectively, and had significantly decreased *Cyclin D1* gene mRNA and protein expression levels, with the *P* values of 0.000 and 0.000, respectively, and there was a significant difference (*P* < 0.05); compared with the blank control group and RhoA-NC group, the RhoA overexpression group had decreased Bax gene mRNA and protein expression levels, with the *P* values of 0.001 and 0.001, respectively, and had increased *Cyclin D1* gene mRNA and protein expression levels, with the *P* values of 0.000 and 0.000, respectively, and there was a significant difference (*P* < 0.05). These were shown in Figures 6 and 7.

### 4.6 Expression levels of vascular endothelial growth factor and transforming growth factor β1 in cells of each group

**Figure 8:**
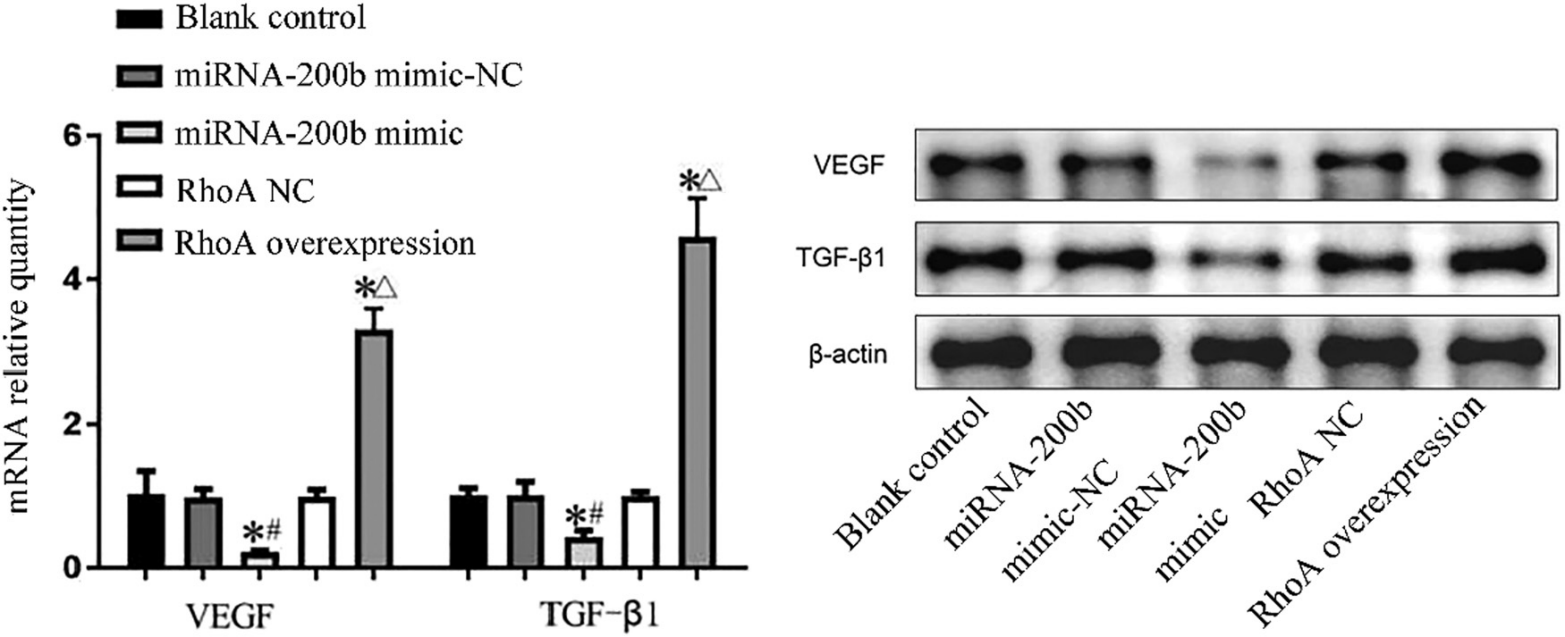
VEGF and TGF-β1 gene mRNA and protein expression levels in each group. Note: compared with blank control group, ^*^ *P* < 0.05; compared with miRNA-200b mimic-NC group, ^#^ *P* < 0.05; and compared with RhoA NC group, ^Δ^ *P* < 0.05.

Compared with the blank control group and the mimic-NC group, the miRNA-200b mimic group had significantly decreased *VEGF* gene mRNA and protein expression levels, with the *P* values of 0.001 and 0.001, respectively, and had significantly decreased *TGF-β1* gene mRNA and protein expression levels, with the *P* values of 0.021 and 0.021, respectively, and there was a significant difference (*P* < 0.05); compared with the blank control group and RhoA-NC group, the RhoA overexpression group had significantly increased *VEGF* gene mRNA and protein expression levels, with the *P* values of 0.000 and 0.000, respectively, and had significantly increased *TGF-β1* gene mRNA and protein expression levels, with the *P* values of 0.000 and 0.000, respectively, and there was a significant difference (*P* < 0.05). This was shown in Figure 8.

## 5 Discussions

Cervical cancer is a common gynecologic malignant tumor and the main cause of cancer-related deaths in women all over the world, especially in developing countries. Therefore, it is required to deeply understand the key genes involved in the occurrence and development of cervical cancer and to discuss its molecular mechanism. In recent years, a large number of studies demonstrated that miRNA regulates gene expression by negatively regulating the stability or translation efficiency of target genes after transcription, so as to play a carcinogenic or tumor suppressor effect in organisms [10]. The miRNA-200b family can inhibit tumors in various human cancers [11]. The abnormal expression of miRNA-200b is of great significance in epithelial–mesenchymal transition (EMT) and transfer of breast carcinoma, colorectal cancer, and glioma cells [12,13]. RhoA was abnormally expressed in various human cancers and involved in the occurrence, development, invasion, and metastasis [14,15]. Teng and Yi-Xue [16] found that RhoA is a downstream target gene of miR-200b, and miR-200b can regulate the blood–tumor barrier permeability by directly targeting RhoA. In this study, HeLa cells of human cervical cancer were taken, and bioinformatics software was adopted to verify the target relationship between miRNA-200b and RhoA. The results indicated that miRNA-200b and RhoA gene 3′UTR have complementary binding sites, and RhoA expression level significantly decreased after miRNA-200b mimics were transfected. These results demonstrated that RhoA is a downstream target gene of miRNA-200b and may be involved in the occurrence and development of cervical cancers.

The miRNAs can inhibit the transaction and translation of mRNA 3′UTR through the targeting function, so as to play the functions of regulating cell proliferation, apoptosis, and transfer [17]. Peng et al. [18] stated that miRNA-200b can inhibit the invasive ability of glioma cells through the targeted regulation of the expression of PROM1. Jin et al. [19] found that miRNA-144 significantly inhibited the proliferation, migration, and invasion of osteosarcoma cells *in vitro* by dual targeting RohA and ROCK1 and inhibited tumor growth and metastasis *in vivo*. The process of tumor occurrence and development is complex. Under normal conditions, the cell cycle and survival-related molecules change due to the inactivation of antioncogene and the activation of oncogenes. VEGF is a specific binding factor for vascular endothelial cells and a key-stimulating factor to promote tumor angiogenesis [20]. TGF-β1 is a negative cellular immunoregulatory factor with dual-directional regulatory function, which plays an important negative regulatory role in the immune escape and immune suppression of tumor cells [21,22]. Studies demonstrated that serum VEGF levels in patients with malignant tumors increased, and cellular immune function was abnormally weakened; the higher the VEGF level is, the more severe the condition of patients with malignant tumors is and the higher the risk of metastasis and invasion is [23]. It was found in some studies that the increased expression of TGF-β1 and Foxp3 proteins in cervical cancer tissues may play an important role in promoting the immune escape of cervical cancer cells and the formation and metastasis of cervical cancer [24,25]. miRNAs are considered to be important molecules for posttranscriptional regulation of gene expression, and they are differentially expressed in tumors of different origins. They not only have tumor suppressor and carcinogenic potential but also have immunomodulatory functions [26]. Studies have reported that miRNA can participate in the immune escape of cancer cells, so that tumor cells are prevented from apoptosis and proliferate indefinitely [27]. Luo et al. [28] found that miRNA-200b may inhibit the invasion of non-small-cell lung cancer cells by targeting VEGF. Ladak et al. [29] stated that miRNA-200b can regulate the expression of TGF-β1 to protect airway epithelial cells from EMT. Therefore, studying the molecular mechanism of tumor proliferation and migration from the perspective of miRNA participating in the immune escape of tumor cell can provide an effective theoretical basis for the treatment of tumor metastasis. The results of this study demonstrated that after transfection of miRNA-200b mimics, the expression level and the cell proliferation ability of VEGF and TGF-β1 genes in HeLa cells significantly decreased, while the level of apoptosis increased; after the overexpression of RhoA gene expression, the expression level and the cell proliferation ability of VEGF and TGF-β1 genes significantly increased, and the level of apoptosis decreased. It is suggested that miRNA-200b inhibits the proliferation ability and promotes the apoptosis of HeLa cells by targeting RhoA and may inhibit the development of cervical cancer by inhibiting the immune escape of cervical cancer cells.

In summary, miRNA-200b can inhibit the proliferation and promote the apoptosis of HeLa cells through the targeted regulation of RhoA; its mechanism of action may be that miRNA-200b inhibits the immune escape of cervical cancer cells. Therefore, it provides an important basis for the treatment of cervical cancer by miRNA-200b from the perspective of immune escape of tumor cells, but its specific mechanism of action needs to be further studied.
